# Spigelian-Cryptorchidism Syndrome in Pediatrics: Diagnostic Value of Ultrasound and Magnetic Resonance Imaging

**DOI:** 10.7759/cureus.88855

**Published:** 2025-07-27

**Authors:** José Luis Herrera Alanís, Oscar Antonio Regalado Morales, Marcelo Valdés Hernández, Amaury Valdés Mancha, Samuel Iván Espinoza Tristán

**Affiliations:** 1 Radiology, Hospital Regional Instituto de Seguridad y Servicios Sociales de los Trabajadores del Estado (ISSSTE), Monterrey, MEX

**Keywords:** abdominal mri, cryptorchidism, ectopic testis, spigelian-cryptorchidism syndrome, spigelian hernia, testicular ultrasound

## Abstract

The Spigelian hernia-cryptorchidism syndrome is a rare clinical entity observed in male neonates. It is characterized by a congenital defect in the Spigelian fascia, resulting in a hernia along the semilunar line, often associated with intestinal obstruction. A hallmark feature is the presence of an ectopic testis, typically located within or immediately adjacent to the hernia sac. This association suggests a developmental anomaly involving both the abdominal wall structures and the genitourinary system. We present the case of a male infant with a history of surgically corrected Tetralogy of Fallot, in whom multiple congenital anomalies were identified, including sacral agenesis type IV, spina bifida, tethered cord, and a horseshoe kidney. During evaluation, a Spigelian hernia containing an undescended left testis was detected, along with a retractile right testis. The diagnosis was initially made by ultrasound and subsequently confirmed by magnetic resonance imaging (MRI), allowing for detailed assessment of the associated anomalies and precise surgical planning. The patient underwent successful surgical management with a favorable outcome. This case illustrates the diagnostic value of imaging, particularly ultrasound and MRI, in identifying rare and complex associations of congenital anomalies, and it highlights the importance of a multimodal imaging approach in guiding timely and effective treatment.

## Introduction

Spigelian-cryptorchidism syndrome is an exceptionally rare clinical entity characterized by the coexistence of a congenital Spigelian hernia and an undescended testis, typically on the same side. The pediatric literature on this condition is sparse, with fewer than 60 cases reported up to 2021 [1], and only a limited number of additional case reports published in the last decade [2,3]. A recent review identified approximately 123 patients with congenital Spigelian hernias, of which only a small subset were associated with undescended testes, often described as isolated case reports [4]. The scarcity of published data, particularly those emphasizing diagnostic imaging, underscores the relevance of our case, which highlights the role of ultrasound and magnetic resonance imaging (MRI) in the accurate diagnosis and surgical planning of this rare syndrome [5].

Sacral agenesis, spina bifida, tethered cord syndrome, and horseshoe kidney are complex congenital abnormalities that, when present in conjunction with an abdominal hernia and cryptorchidism, reflect a profound disruption in embryological development affecting multiple systems [6].

We present the case of a male infant with a history of surgically corrected Tetralogy of Fallot, who was later diagnosed with sacral agenesis type IV according to Renshaw’s classification. This severe form of sacral agenesis is characterized by complete absence of the sacrum, with the iliac bones articulating directly with the lowest lumbar vertebra. The patient also presented with multiple associated congenital anomalies, including spina bifida, tethered cord, and a horseshoe kidney. Additionally, a Spigelian hernia was identified, containing an undescended left testis, while the right testis was retractile.

This case underscores the importance of early diagnosis and coordinated multidisciplinary management in patients with complex congenital anomalies. The delayed identification of the undescended left testis, ultimately located within the Spigelian hernia sac, resulted in testicular atrophy and the need for orchiectomy. Undescended testes are associated with an increased risk of infertility and malignancy, particularly when not addressed in a timely manner. Early imaging, specifically ultrasound and MRI, could have enabled prompt recognition of the hernia and its contents, facilitating earlier surgical intervention and potentially preserving testicular function [7]. A structured, collaborative approach involving radiology, pediatric surgery, and urology is essential to ensure accurate diagnosis, comprehensive evaluation, and optimal outcomes in such rare and complex presentations. 

## Case presentation

A 15-month-old male infant, born via cesarean section at 37 weeks of gestation to a 25-year-old mother, was referred with a palpable abdominal mass and an undescended left testis. His mother had three pregnancies, all delivered by cesarean section, and had no history of vaginal birth (G3 P0 C3). She had no relevant medical history, and the patient’s siblings were healthy. At birth, the patient had Apgar scores of 5-9, and a Silverman-Anderson (SA) score of 0-0. Initial steps of neonatal resuscitation were provided, including two cycles of positive pressure ventilation (PPV), which resulted in recovery of heart rate. The patient had a history of Tetralogy of Fallot, which was surgically corrected shortly after birth, along with multiple congenital anomalies involving the nervous and genitourinary systems, including a tethered cord, neurogenic bladder, horseshoe kidney, spina bifida, and type IV sacral agenesis according to Renshaw’s classification.

**Figure 1:**
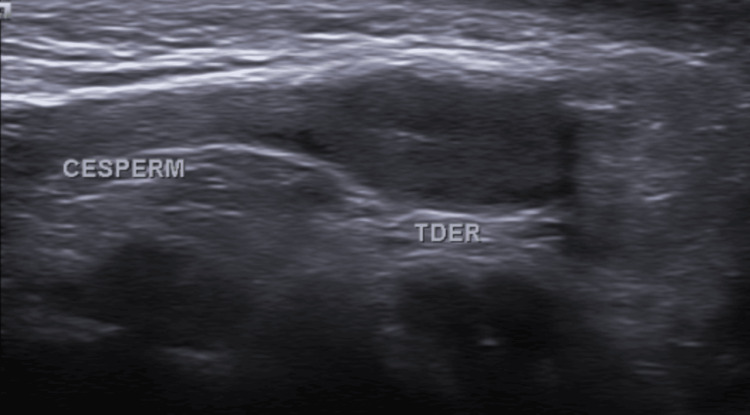
Grayscale testicular ultrasound The right testis is absent from the scrotal sac; it is identified in the inguinal region, compatible with a retractile testis

**Figure 2:**
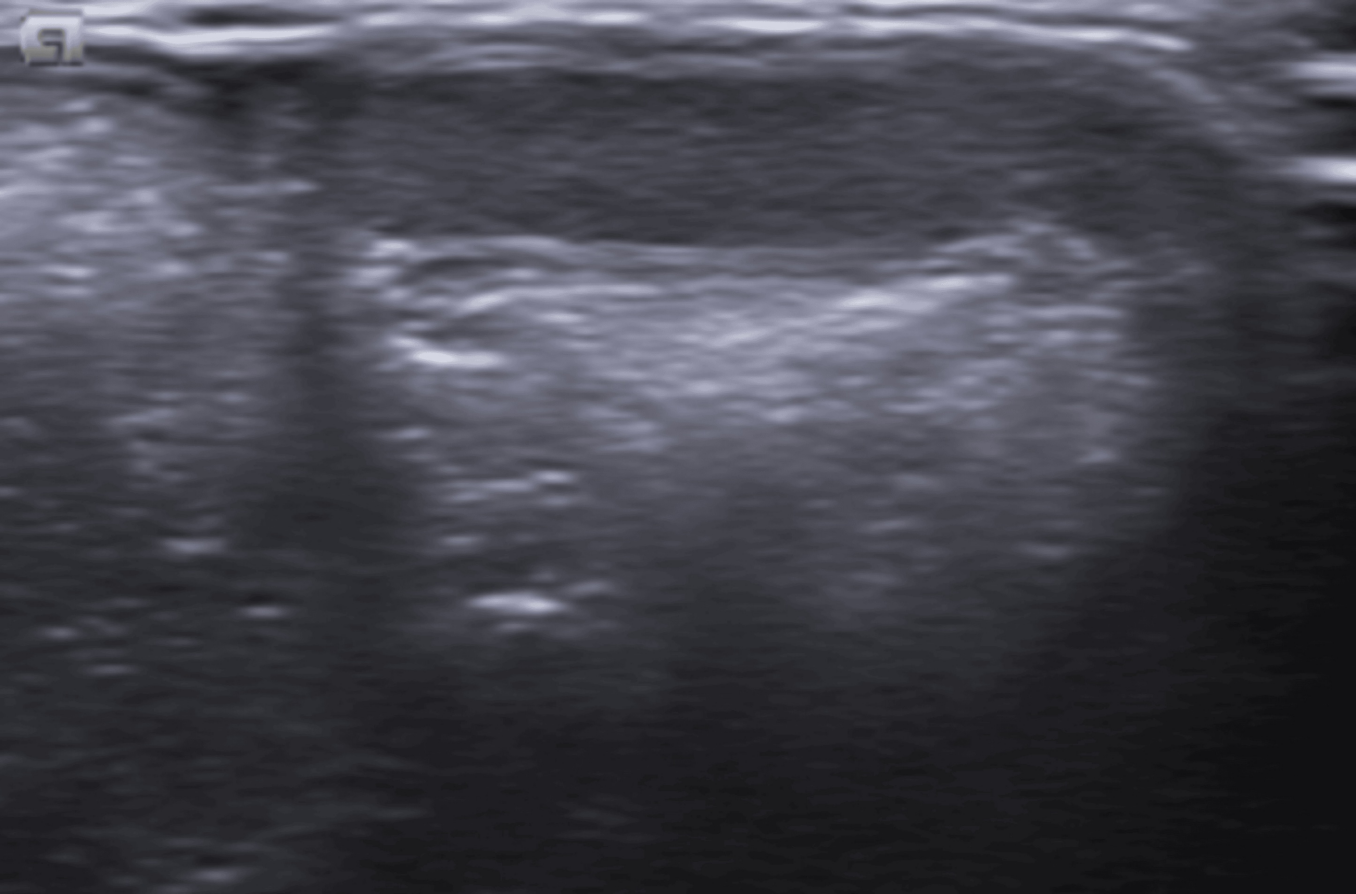
Grayscale abdominal wall ultrasound A defect in the abdominal wall is identified in the left lower quadrant, with herniation of a sac containing an ovoid structure compatible with a testis. The testis demonstrates decreased echogenicity and size compared to expected, suggesting testicular atrophy

Clinical examination revealed an abdominal wall hernia in the left lower quadrant, associated with left-sided cryptorchidism and a retractile right testis (Figure 1). Ultrasound images confirmed the presence of an abdominal wall defect consistent with a Spigelian hernia, suggesting the presence of an ectopic left testis (Figure 2).

**Figure 3:**
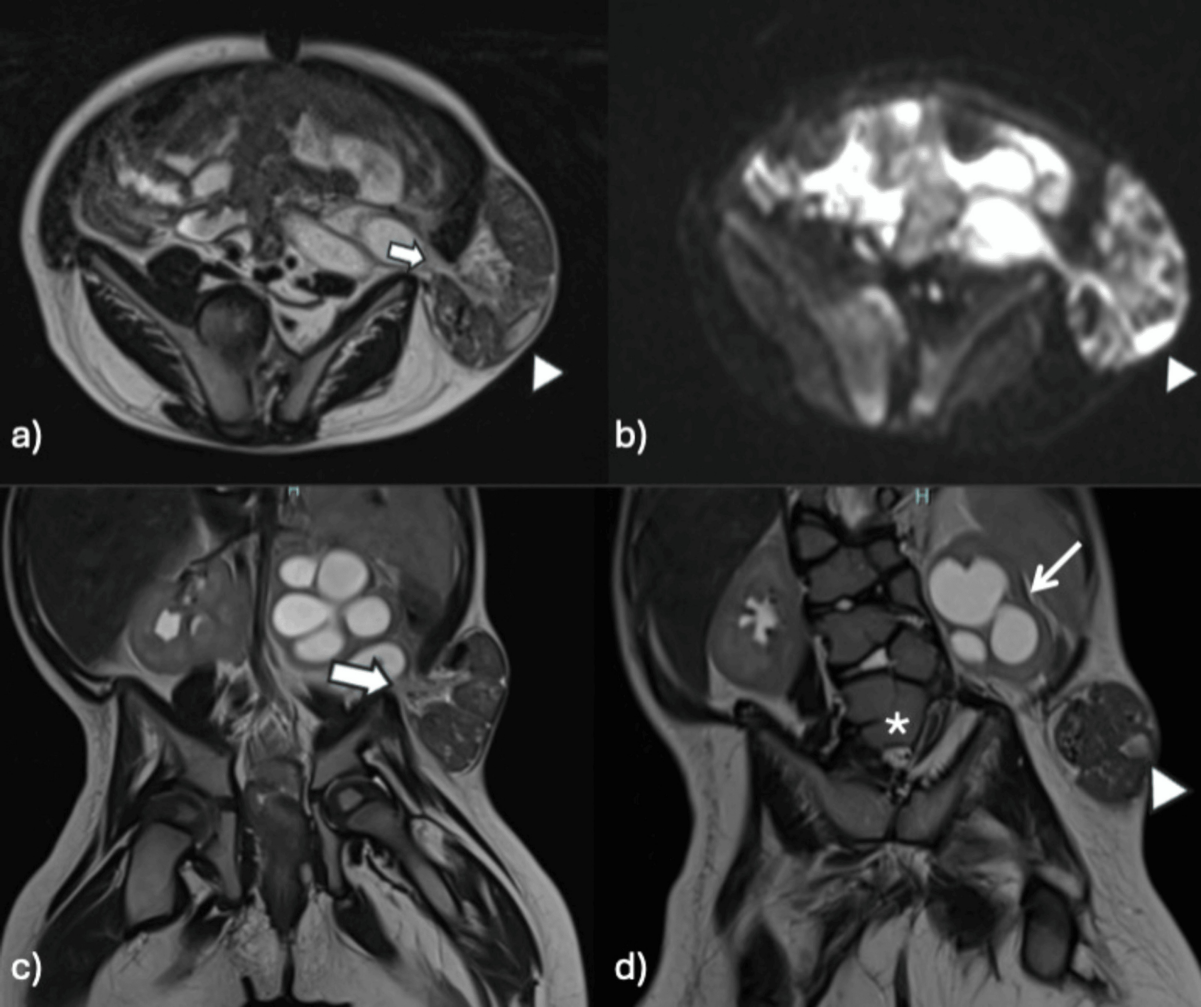
MRI axial and coronal sections (a) T2-weighted MRI, axial section, showing a defect (arrow) in the right lateral abdominal wall with the formation of a hernial sac containing intestinal contents, identifying an image inside compatible with a left testicle (arrowhead). (b) Diffusion-weighted MRI shows restricted diffusion of the left testicle (arrowhead). Although the corresponding ADC map demonstrated low signal intensity, confirming true diffusion restriction (not shown). (c) and (d) T2-weighted MRI in coronal sections, showing a defect (thick arrow) in the abdominal wall with the formation of a hernial sac containing intestinal contents, identifying a testicle inside (arrowhead). Additionally, previously known findings are noted, such as left hydronephrosis (thin arrow) and butterfly-shaped lumbar vertebral bodies (asterisk). ADC: apparent diffusion coefficient

**Figure 4:**
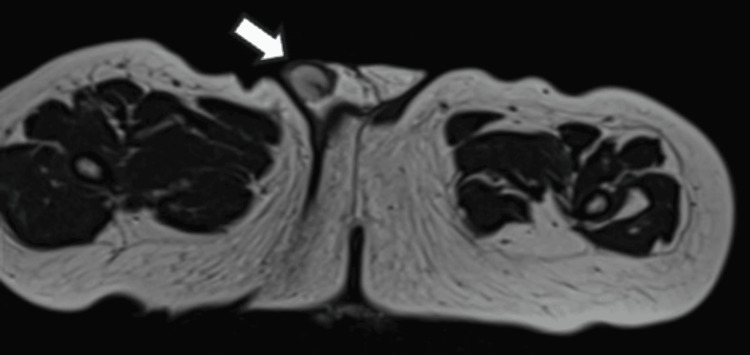
Axial T2-weighted MRI Right testicle (arrow) at the level of the scrotal sac

Following the initial ultrasound evaluation, which identified the undescended left testis within the Spigelian hernia, an MRI was performed to confirm the diagnosis and further delineate the anatomical relationships of the hernia sac and testicular structures (Figures 3, 4).

MRI identified the left testis within the palpable hernia sac, confirming the diagnosis of Spigelian-cryptorchidism syndrome.

At 21 months of age, the patient underwent surgical intervention. The two-week interval between imaging and surgery allowed for adequate preoperative planning and coordination among the pediatric surgery and urology teams. Despite timely diagnosis and intervention, the left testis was found to be atrophic during surgery, necessitating orchiectomy. This outcome underscores the importance of early imaging and prompt referral to surgical care to avoid irreversible testicular damage.

## Discussion

Cryptorchidism is one of the most common congenital anomalies in male infants, with an incidence of up to 3% in full-term and up to 30% in preterm newborns [7]. While it often presents as an isolated finding, its occurrence in association with other congenital anomalies, particularly genitourinary and neurological malformations, warrants a thorough evaluation. In this case, the presence of a Spigelian hernia containing an undescended testis, along with a retractile contralateral testis, further complicates the clinical picture.

Spigelian hernias are rare abdominal wall defects, accounting for less than 2% of all hernias [6]. Their association with undescended testes is uncommon but has been previously reported in pediatric populations [8]. The coexistence of these two conditions may suggest a shared embryological disruption in the development of the abdominal wall and the gubernacular pathway [9]. Timely recognition of this association is critical, as delay in treatment increases the risk of testicular atrophy, infertility, and potential malignancy [10].

Moreover, this patient presented with a complex spectrum of congenital anomalies, including corrected Tetralogy of Fallot, sacral agenesis type IV, spina bifida, tethered cord, and horseshoe kidney, all of which are associated with varying degrees of embryonic mesodermal and neuroectodermal dysgenesis. The presence of a tethered cord is particularly significant, as it has been linked to genitourinary dysfunction, including cryptorchidism and bladder/bowel control issues. This supports the hypothesis that neurologic abnormalities may interfere with normal testicular descent either directly through impaired innervation or indirectly via altered intra-abdominal pressures [11].

Imaging played a pivotal role in this case. Ultrasound was essential in identifying testicular location and viability, while MRI allows for the exact localization of the testicle outside the scrotum, can evaluate its viability, and determines the presence of complications; a viable testicle without complications typically presents with a normal or moderate signal on diffusion-weighted imaging and higher apparent diffusion coefficient values, which helps determine the best therapeutic approach, as well as anatomical assessment, particularly in the evaluation of the tethered cord and hernia contents. The comprehensive use of imaging facilitated a multidisciplinary approach to management, involving pediatric surgery, urology, radiology, and neurosurgery [12].

## Conclusions

This case illustrates the complex interplay between multiple congenital anomalies, including cardiac, neural tube, genitourinary, and abdominal wall defects. The identification of a Spigelian hernia containing an undescended testis in a patient with sacral agenesis, tethered cord, and horseshoe kidney underscores the importance of maintaining a high index of suspicion and pursuing a comprehensive imaging evaluation. In male infants presenting with cryptorchidism and a palpable abdominal wall mass, Spigelian hernia should be considered, as this rare association can lead to delayed diagnosis and irreversible complications such as testicular atrophy, infertility, or malignancy.

Prompt ultrasound evaluation is essential as a first-line imaging modality to identify the hernia and assess testicular location. If ultrasound findings raise concern for an ectopic testis or hernia, MRI should be performed without delay to confirm the diagnosis, evaluate testicular viability, particularly using DWI and ADC sequences, and guide surgical planning. Early recognition and coordinated multidisciplinary management are key to optimizing outcomes in these rare and complex cases. Clinicians should always consider the possibility of coexisting anomalies in patients with known congenital conditions and tailor the diagnostic approach accordingly.
